# Epigenetic clock analysis of blood samples in drug-naive first-episode schizophrenia patients

**DOI:** 10.1186/s12888-023-04533-1

**Published:** 2023-01-17

**Authors:** Zongchang Li, Xiaofen Zong, David Li, Ying He, Jinsong Tang, Maolin Hu, Xiaogang Chen

**Affiliations:** 1 Department of Psychiatry, and National Clinical Research Center for Mental Disorders, The Second Xiangya Hospital, Central South University, No 139 Renmin Road, Changsha, Hunan 410011 P. R. China; 2 China National Technology Institute on Mental Disorders & Hunan Key Laboratory of Psychiatry and Mental Health, Mental Health Institute of the Second Xiangya Hospital, Central South University, Changsha, P. R. China; 3 Department of Psychiatry, Renmin Hospital of Wuhan University, Wuhan, P. R. China; 4 Department of Psychiatry, Sir Run Run Shaw Hospital, Zhejiang University School of Medicine, Hangzhou, P. R. China

**Keywords:** Schizophrenia, DNA methylation, Epigenetic age, Antipsychotic treatment

## Abstract

**Background:**

Schizophrenia (SCZ) is a severe and chronic psychiatric disorder with premature age-related physiological changes. However, numerous previous studies examined the epigenetic age acceleration in SCZ patients and yielded inconclusive results. In this study, we propose to explore the epigenetic age acceleration in drug-naive first-episode SCZ (FSCZ) patients and investigate whether epigenetic age acceleration is associated with antipsychotic treatment, psychotic symptoms, cognition, and subcortical volumes.

**Methods:**

We assessed the epigenetic age in 38 drug-naive FSCZ patients and 38 healthy controls by using three independent clocks, including Horvath, Hannum and Levine algorithms. The epigenetic age measurements in SCZ patients were repeated after receiving 8 weeks risperidone monotherapy.

**Results:**

Our findings showed significantly positive correlations between epigenetic ages assessed by three clocks and chronological age in both FSCZ patients and healthy controls. Compared with healthy controls, drug-naive FSCZ patients have a significant epigenetic age deceleration in Horvath clock (*p* = 0.01), but not in Hannum clock (*p* = 0.07) and Levine clock (*p* = 0.43). The epigenetic ages of Hannum clock (*p* = 0.002) and Levine clock (*p* = 0.01) were significantly accelerated in SCZ patients after 8-week risperidone treatment. However, no significant associations between epigenetic age acceleration and psychotic symptoms, cognitive function, as well as subcortical volumes were observed in FSCZ patients.

**Conclusion:**

These results demonstrate that distinct epigenetic clocks are sensitive to different aspects of aging process. Further investigations with comprehensive epigenetic clock analyses and large samples are required to confirm our findings.

**Supplementary Information:**

The online version contains supplementary material available at 10.1186/s12888-023-04533-1.

## Background

Schizophrenia (SCZ) is one of the most disabling illnesses [1], affecting approximately 1% of the general population worldwide. SCZ patients have a higher rate of premature mortality [2], and their life expectancy is roughly 15 years less than healthy individuals [3]. Though suicides and accidents account for a large portion of premature mortality in SCZ, the majority of morbidity is attributed to age-related diseases, such as diabetes [4], cardiovascular disease [5, 6], and cancer [7].

Multiple lines of evidence suggest that patients with SCZ show premature aging characteristics, such as cognitive decline [8, 9], dendritic spine loss [10], cortical atrophy [11], shorted telomere [12], and increased levels of inflammatory factors and oxidative stress [13]. The accelerated aging hypothesis of SCZ has thus been proposed as a cause for the excess mortality in SCZ. This hypothesis proposes SCZ as a syndrome of accelerated aging associated with premature physiological change that increases the risk of aging-related medical conditions and mortality [14]. However, testing this hypothesis is difficult due to the lack of accurate and robust biomarkers for biological age.

The recent development of DNA methylation (DNAm) based epigenetic clocks (also called epigenetic ages) offers a promise for addressing this challenge. The epigenetic age was integrated with DNAm levels at a set of cytosine-phosphate-guanine (CpG) sites using mathematical algorithms, producing an accurate and well-validated measure of chronological age [15]. Epigenetic age acceleration or deceleration is defined as an increase or decrease in epigenetic age compared to chronological age. Accumulating evidence has shown that epigenetic age acceleration is associated with numerous risk factors and outcomes for psychiatric disorders, including adversity exposure [16–18], cognitive decline [19–21], altered brain structures [22–25], and depression [26–28]. However, previous studies have shown inconsistent results in SCZ. Although no acceleration of epigenetic aging has consistently been reported in blood and brain tissues of patients with SCZ [29, 30], some studies also have detected accelerated or delayed epigenetic aging in patients with SCZ [31–35]. Considering the pathological and clinical heterogeneity of SCZ, confounding variables such as antipsychotic medications and illness duration may partially explain these inconsistent findings.

Here, we investigated the epigenetic age in a cohort of drug-naive FSCZ patients and healthy controls using three independent epigenetic clock approaches, including Horvath [36], Hannum [37] and Levine [38] methods. All drug-naive FSCZ patients received treatment with risperidone monotherapy and were followed up for 8 weeks. Then, we assessed the effect of antipsychotic treatment on the epigenetic aging process in FSCZ and investigated the potential associations of epigenetic age acceleration with psychotic symptoms, cognitive function, as well all subcortical volumes.

## Methods

### Participants

Our study recruited 38 drug-naive FSCZ patients from the Henan Mental Hospital. Diagnosis of schizophrenia were established by two experienced psychiatrists based on the criteria from the DSM-IV-TR while only individuals with an initial onset of SCZ with no medical history of antipsychotic medication or psychotropic drugs use were selected. A monotherapy session consisting of risperidone for 8 weeks were applied to the FSCZ subjects with a follow-up at the end of treatment. Additionally, thirty-eight healthy volunteers with no previous history of psychiatric or neurological disorders were recruited from the local community to serve as the healthy controls. This study was approved by the Ethics Committee of the Second Xiangya Hospital of Central South University. All the participants provided written informed consent after receiving a complete study description.

### Psychopathological and neurocognitive function assessments

The severity of psychotic symptoms of the FSCZ patients at baseline and after 8 weeks of treatment were assessed by using the 30-item Positive and Negative Symptom Scale (PANSS). The improvement of antipsychotic treatment at 8 weeks was evaluated by the percentage change in PANSS as described previously [39].

The cognitive functions of participants were analyzed by utilizing the Digit Span Tests (DST-Forward and DST-Backward), the Verbal Fluency Test (VFT), the Stroop Tests (Stroop-W: words, Stroop-C: colours, and Stroop-I: interference), the Trail Making Test (TMT-part A and TMT-part B), and the Wisconsin Card Sorting Test (WCST-C: categories and WCST-P: perseverative errors, 128 cards). The cognitive function of all participants was evaluated at baseline while the 36 FSCZ patients were reassessed after an eight-week follow-up. Cognitive function improvement in FSCZ patients before and after treatment was determined by the differences in cognitive function scores between 8-week follow-up and baseline.

### Imaging data acquisition and processing

All participants were scanned with a Siemens 3.0 T MRI scanner (Verio). Left and right accumbens, amygdala, caudate, hippocampus, pallidum, putamen, thalamus, as well as intracranial volume (ICVs), were obtained from high-resolution T1-weighted structural brain data by using the programme’s segmentation procedure of FreeSurfer software package (v5.3.0, http://surfer.nmr.mgh.harvard.edu). Details on acquisition parameters and image processing are described in our previous study [40].

### Genome-wide methylation analysis

Genomic DNA from all participants was extracted from whole blood samples by using the QIAamp DNA Blood Mini Kit (QIAGEN), followed by bisulfite conversion with EZ DNA Methylation™Kit (Zymo Research; Irvine, CA). For each sample, the genome-wide DNA methylation was measured by using the Infinium HumanMethylation450 BeadChip (Illumina, San Diego, CA), and the β-values of DNA methylation were calculated and normalized by using the GenomeStudio software(v1.9). Full details of the DNAm analysis are described in our previous paper [41].

### Epigenetic age estimates

Epigenetic age was calculated using three independent epigenetic clocks: Horvath, Hannum and Levine algorithms. Horvath’s epigenetic age was trained on chronological age by using a total of 353 CpG sites across tissues and cell types [36]. Hannum’s epigenetic age was trained on chronological age based on 71 CpG probes from whole blood samples [37]. Levine algorithm trained on 9 aging biomarkers and chronological age using 513 CpG probes from blood samples, and developed an epigenetic biomarker of aging, called “DNAm PhenoAge” [38]. Horvath’s epigenetic age was estimated with the online DNA Methylation Age Calculator (https://dnamage.genetics.ucla.edu/). Epigenetic ages for Hannum and Levine’s clocks were estimated based on the coefficients of CpG sites listed in Hannum et al’s [37] and Levine et al’s papers [38]. For each epigenetic clock, the epigenetic age acceleration was defined as AgeAccelerationResidual (AgeAccel) and was calculated by using the residual from regressing epigenetic age on chronological age.

### Statistical analyses

For continuous variables, Pearson’s or Spearman’s rank correlation analyses were conducted to assess their relationships. Differences in epigenetic age accelerations between drug-naive FSCZ patients and healthy controls at baseline were compared by using multiple linear regression analyses after adjusting for sex, age, and tobacco use. Changes in epigenetic age acceleration in drug-naive FSCZ patients at baseline and after 8-week treatment were tested with Paired samples t-test. All data were analyzed using the Statistical Package for Social Sciences (SPSS, version 18.0).

## Results

### Descriptive characteristics of the study participants

The cohort of our study consists of 38 drug-naive FSCZ patients (25 male and 13 female; mean age ± SD, 25.00 ± 4.95 years) and 38 healthy controls (25 male and 13 female; 24.76 ± 4.56 years). No differences were observed in chronological age and sex between FSCZ patients and controls (*p* > 0.05). The demographic and clinical characteristics of the samples are shown in the Supplemental Table 1.

### Accuracy of epigenetic age estimates

**Fig. 1:**
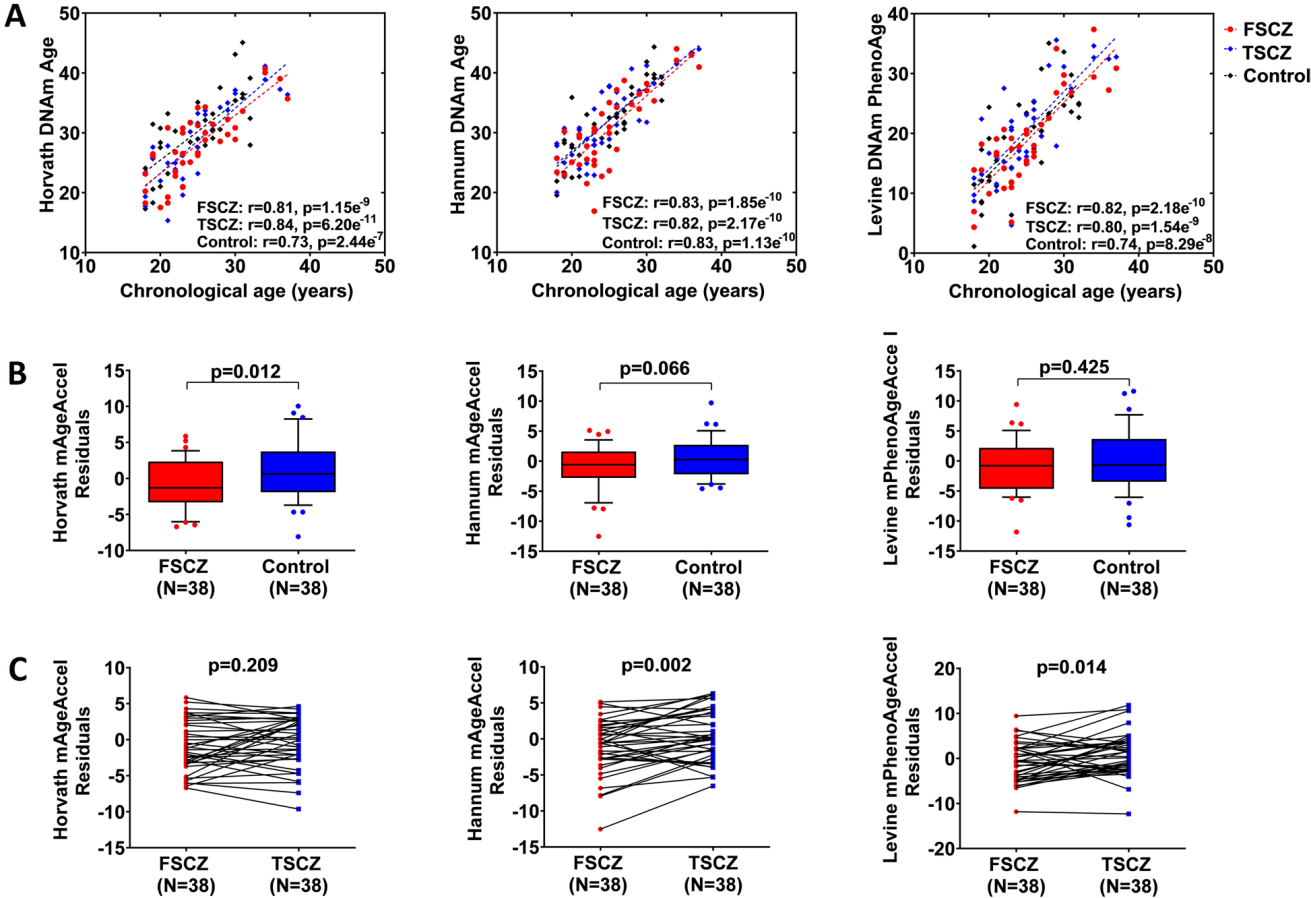
Epigenetic age analyses in drug-naive first-episode schizophrenia (SCZ) patients and healthy controls. **A** Significant Pearson’s correlations between epigenetic age and chronological age in SCZ patients at baseline (red) and after 8 weeks treatment (blue), and healthy controls (black). Each panel of the figure shows scatter plots of epigenetic age (y-axis) against chronological age (x-axis) in whole blood sample measured by Horvath, Hannum, and Levine epigenetic clocks. **B** Comparison of epigenetic age acceleration between drug-naive first-episode SCZ patients and healthy controls. Between-group difference of epigenetic age acceleration measured by Horvath, Hannum, and Levine epigenetic clocks were tested by regression analyses adjusting for age, sex, and tobacco use. **C** Antipsychotic effects on epigenetic age acceleration in drug-naive first-episode SCZ patients. The changes of epigenetic age acceleration in drug-naive first-episode SCZ patients before and after 8-week risperidone treatment were tested with Paired samples t-test. FSCZ: drug-naive first-episode schizophrenia, TSCZ: 8-week antipsychotics treated drug-naive first-episode schizophrenia

As expected, chronological age has strong positive correlations with Horvath’s epigenetic age (*rho* = 0.79, *p* = 1.55e^− 25^), Hannum’s epigenetic age(*rho* = 0.82, *p* = 2.14e^− 28^), and Levine DNAm PhenoAge (*rho* = 0.79, *p* = 9.79e^− 26^) in all subjects. The significant correlations did not change when patients and controls were analyzed separately (Fig. 1A). Compared with chronological age, Horvath and Hannum’s epigenetic ages were overestimated by 4.0 and 6.6 years respectively, and Levine’s DNAm PhenoAge was underestimated by 5.4 years. Comparing AgeAccel among these three epigenetic clocks using all samples, we found a moderate concordance (r = 0.293–0.541), indicating a substantial variation in the epigenetic age acceleration estimated by these three clocks.

### Comparison of epigenetic age acceleration between FSCZ patients and healthy controls

Multiple regression analyses found a significant difference between FSCZ patients and healthy controls in Horvath’s AgeAccel (β = − 0.28, *p* = 0.01), but not in Hannum’s AgeAccel (β = − 0.21, *p* = 0.07) and Levine’s PhenoAgeAccel (β = − 0.09, *p* = 0.42) (Fig. 1B). The mean Horvath’s AgeAccel in FSCZ patients and controls are − 0.93 and 1.25, indicating a deceleration with 2.18 years in FSCZ patients compared to controls. In addition, multiple regression analyses revealed a sex-effect on Horvath’s AgeAccel (β = − 0.29, *p* = 0.01) and Horvath’s AgeAccel (β = − 0.25, *p* = 0.03). No age effect on epigenetic age acceleration was observed for all three epigenetic clocks (*p* > 0.05).

### Antipsychotic effect on epigenetic age acceleration

Paired samples t-test analyses showed significant changes of Hannum’s AgeAccel (t = − 3.30, *p* = 0.002) and Levine’s PhenoAgeAccel (t = − 2.59, *p* = 0.01) in FSCZ patients before and after 8-week risperidone treatment (Fig. 1C). The mean Hannum’s AgeAccel and Levine’s PhenoAgeAccel in SCZ patients were − 1.02 and − 0.92 at baseline and 0.53 and 0.94 after treatment, suggesting that Hannum’s and Levine’s epigenetic ages are respectively accelerated with 1.55 and 1.86 years in FSCZ patients after 8-week risperidone treatment. For Horvath’s AgeAccel, an acceleration of 0.61 years was observed in FSCZ patients although the change is not statistically significant (t = − 1.28, *p* = 0.21). Multiple regression analyses found that there were no significant differences in the epigenetic age acceleration for all three epigenetic clocks (*p* > 0.05) between FSCZ patients after 8 weeks risperidone treatment and healthy controls.

### Association of epigenetic age acceleration with psychotic symptom severity and cognitive function

Multiple linear regression analyses were performed to examine the associations between epigenetic age acceleration and PANSS scores in FSCZ patients at baseline as well as the correlation of baseline epigenetic age acceleration with the percentage change in PANSS after 8-week treatment, adjusting for age, sex, tobacco use, and illness duration. Our result showed that epigenetic age accelerations for all three epigenetic clocks have no significant correlations with PANSS scores and PANSS percentage change values (Table 1).

**Table 1:** No correlations of epigenetic aging acceleration estimated by three clocks with the psychosis symptom severity and symptom improvement in patients with drug-naive first-episode SCZ

Valuable	Horvath’s AgeAccel		Hannum’s AgeAccel		Levine’s PhenoAgeAccel	
	β	***P*** value	β	***P*** value	β	***P*** value
Baseline PANSS Scores						
Positive syndromes	−0.02	0.92	0.22	0.23	0.35	0.06
Negative syndromes	−0.03	0.88	0.07	0.69	0.17	0.31
General syndromes	−0.05	0.78	0.07	0.70	0.13	0.47
PANSS total syndromes	−0.05	0.79	0.14	0.42	0.27	0.13
Percentage change in PANSS Scores						
Percentage change in positive syndromes	−0.02	0.92	0.04	0.80	−0.25	0.15
Percentage change in negative syndromes	0.05	0.78	−0.22	0.18	−0.34	0.05
Percentage change in general syndromes	0.07	0.69	0.10	0.56	−0.03	0.85
Percentage change in PANSS total syndromes	0.07	0.67	0.02	0.89	−0.23	0.19

Multiple linear regression analyses were conducted to assess the relationship of baseline epigenetic age acceleration with baseline psychosis symptom severity and symptom improvement after 8-week treatment in SCZ patients adjusting for age, sex, tobacco use, and illness duration

For cognitive function, multiple linear regression analyses revealed no significant relationship between epigenetic age acceleration and cognitive function in all participants at baseline (Table 2). After 8-week treatment, epigenetic age accelerations for all three epigenetic clocks at baseline are negatively correlated with the improvement of Stroop-W in FSCZ patients (*p* < 0.05). Furthermore, Hannum’s AgeAccel and Levine’s PhenoAgeAccel at baseline have significantly negative correlations with the improvement of Stroop-C in FSCZ patients (*p* < 0.05). However, no significant results remain after Bonferroni correction (Table 3).

**Table 2:** No correlations of epigenetic aging acceleration estimated by three clocks with the cognitive function in drug-naive first-episode SCZ and healthy controls

Cognition Valuable	Horvath’s AgeAccel		Hannum’s AgeAccel		Levine’s PhenoAgeAccel	
	β	***P*** value	β	***P*** value	β	***P*** value
DST-F	0.06	0.58	0.06	0.60	− 0.06	0.62
DST-B	0.20	0.09	−0.02	0.90	0.01	0.92
VFT	−0.04	0.74	−0.01	0.94	0.05	0.69
Stroop-W	0.06	0.64	0.04	0.72	0.11	0.35
Stroop-C	−0.06	0.60	0.02	0.88	0.07	0.57
Stroop-I	−0.02	0.88	0.03	0.81	0.17	0.18
TMT-A	−0.08	0.52	−0.17	0.17	0.00	0.98
TMT-B	−0.05	0.68	−0.04	0.74	−0.01	0.91
WCST-C	−0.20	0.11	−0.18	0.14	−0.14	0.24
WCST-PE	0.17	0.16	0.02	0.87	0.10	0.41

*DST-F* Digit Span Tests Forward, *DST-B* Digit Span Tests Backward, *VFT* Verbal Fluency Test, *TMT-A* Trail Making Test-Part A, *TMT-B* Trail Making Test-Part B, *Stroop-W* Stroop Tests Words, *Stroop-C* Stroop Tests Colors, *Stroop-I* Stroop Tests Interference, *WCST-C* Wisconsin Card Sorting Test categories, *WCST-P* Wisconsin Card Sorting Test perseverative errors. Multiple linear regression analyses were conducted to assess the relationship between epigenetic age acceleration and cognitive function in 38 SCZ patients and 38 controls at baseline adjusting for age, sex and tobacco use

**Table 3:** Association of epigenetic aging acceleration estimated by three clocks with the cognitive function improvement in drug-naive first-episode SCZ after 8 weeks resperidone treatment

Cognition Valuable	Horvath’s AgeAccel		Hannum’s AgeAccel		Levine’s PhenoAgeAccel	
	β	***P*** value	β	***P*** value	β	***P*** value
DST-F	0.02	0.92	−0.12	0.50	−0.13	0.48
DST-B	−0.05	0.77	**0.34**	**0.04**	0.04	0.82
VFT	0.09	0.63	0.20	0.27	0.35	0.06
Stroop-W	**−0.34**	**0.04**	**−0.34**	**0.04**	**−0.37**	**0.03**
Stroop-C	−0.16	0.38	**−0.46**	**0.01**	−0.32	0.07
Stroop-I	0.08	0.67	−0.23	0.19	**−0.39**	**0.03**
TMT-A	−0.03	0.87	0.13	0.48	−0.09	0.64
TMT-B	−0.08	0.69	0.03	0.86	0.03	0.89
WCST-C	**0.41**	**0.01**	0.15	0.34	0.08	0.64
WCST-PE	0.12	0.49	−0.01	0.98	0.07	0.71

*DST-F* Digit Span Tests Forward, *DST-B* Digit Span Tests Backward, *VFT* Verbal Fluency Test, *TMT-A* Trail Making Test-Part A, *TMT-B* Trail Making Test-Part B, *Stroop-W* Stroop Tests Words, *Stroop-C* Stroop Tests Colors, *Stroop-I* Stroop Tests Interference, *WCST-C* Wisconsin Card Sorting Test categories, *WCST-P* Wisconsin Card Sorting Test perseverative errors. Multiple linear regression analyses were conducted to assess the relationship between baseline epigenetic age acceleration and improvement of cognitive function in 36 SCZ patients after 8-week treatment adjusting for age, sex, tobacco use, and illness duration. Bold values indicate significant difference (*P* < 0.05) before Bonferroni correction

### Association of epigenetic age acceleration with subcortical volumes

At baseline, epigenetic age acceleration has no significant associations with the volume of 14 subcortical regions in all participants, adjusting for age, sex, tobacco use, and ICV (Table 4). For FSCZ patients, multiple linear regression analyses showed that baseline Horvath’s AgeAccel has a significantly negative correlation with the change of right accumben volume (β = − 0.43, *p* = 0.01), but not remained after Bonferroni correction (Table 5).

**Table 4:** No correlations of epigenetic aging acceleration estimated by three clocks with the subcortical volume in drug-naive first-episode SCZ and healthy controls

Subcortical volumes	Horvath’s AgeAccel		Hannum’s AgeAccel		Levine’s PhenoAgeAccel	
	β	***P*** value	β	***P*** value	β	***P*** value
Thalamus_L	0.10	0.23	0.08	0.37	0.19	0.16
Caudate_L	0.12	0.27	0.19	0.06	−0.07	0.49
Putaman_L	0.00	0.98	0.06	0.63	0.00	1.00
Pallidum_L	0.14	0.22	0.05	0.67	0.00	0.99
Hippocampus_L	−0.05	0.65	0.02	0.84	−0.08	0.43
Amygdala_L	0.05	0.60	−0.08	0.42	−0.12	0.24
Accumbens_L	0.11	0.37	0.04	0.74	−0.07	0.57
Thalamus_R	0.05	0.52	−0.04	0.66	−0.07	0.43
Caudate_R	0.14	0.15	0.19	0.06	−0.04	0.70
Putaman_R	0.04	0.67	0.08	0.43	0.01	0.89
Pallidum_R	0.12	0.31	0.01	0.91	0.05	0.64
Hippocampus_R	−0.02	0.88	−0.02	0.88	−0.06	0.58
Amygdala_R	0.04	0.73	−0.09	0.39	−0.04	0.68
Accumbens_R	−0.04	0.74	0.05	0.69	−0.21	0.06

*L* Left, *R* Right. Multiple linear regression analyses were conducted to assess the relationship between epigenetic age acceleration and 14 subcortical volumes in 38 SCZ patients and 38 controls at baseline adjusting for age, sex, tobacco use, and intracranial volume

**Table 5:** Association of epigenetic aging acceleration estimated by three clocks with the subcortical volume changes in drug-naive first-episode SCZ after 8 weeks resperidone treatment

Subcortical volumes	Horvath’s AgeAccel		Hannum’s AgeAccel		Levine’s PhenoAgeAccel	
	β	***P*** value	β	***P*** value	β	***P*** value
Thalamus_L	−0.30	0.06	−0.14	0.38	−0.09	0.58
Caudate_L	−0.12	0.48	**−0.38**	**0.01**	−0.15	0.36
Putaman_L	0.26	0.11	0.00	0.99	−0.07	0.65
Pallidum_L	−0.01	0.93	0.05	0.74	0.11	0.53
Hippocampus_L	0.13	0.51	−0.12	0.49	−0.30	0.10
Amygdala_L	0.15	0.42	0.01	0.97	−0.18	0.32
Accumbens_L	−0.23	0.22	−0.04	0.82	−0.04	0.83
Thalamus_R	0.06	0.74	0.15	0.38	0.09	0.60
Caudate_R	−0.03	0.86	− 0.23	0.19	0.02	0.90
Putaman_R	−0.12	0.51	−0.15	0.40	−0.16	0.37
Pallidum_R	−0.25	0.18	0.10	0.57	0.09	0.63
Hippocampus_R	−0.21	0.58	−0.08	0.66	−0.06	0.73
Amygdala_R	−0.15	0.43	−0.05	0.80	0.11	0.55
Accumbens_R	**−0.43**	**0.01**	−0.15	0.41	−0.02	0.94

*L* Left, *R* Right. Multiple linear regression analyses were conducted to assess the relationship between baseline epigenetic age acceleration and changes of subcortical volumes in 38 SCZ patients after 8-week treatment adjusting for age, sex, illness duration, tobacco use, and intracranial volume. Bold values indicate significant difference (*P* < 0.05) before Bonferroni correction

## Discussion

This study investigated the epigenetic age and the effect of antipsychotic treatment on the epigenetic aging process in drug-naive FSCZ patients. We measured the epigenetic age acceleration by using three independent algorithms, including Horvath, Hannum and Levine’s epigenetic clocks. Our findings demonstrated a significant epigenetic age deceleration in Horvath’s epigenetic clock among drug-naive FSCZ patients relative to controls. In addition, we found that epigenetic aging of Hannum and Levine clocks was significantly accelerated in patients with FSCZ after 8-week risperidone treatment.

This study showed that epigenetic age acceleration is delayed in patients with drug-naive FSCZ against the accelerated aging hypothesis of SCZ. Our finding is consistent with two recent studies [33, 42]. Talarico et al. found longer telomere length and decreased epigenetic age in drug-naive FSCZ patients [42]. Wu et al. revealed an epigenetic age deceleration in SCZ patients by using the largest size of methylation datasets from 1211 brain tissues and 2333 whole blood samples [33]. These results support the hypothesis that SCZ may be a neurodevelopmental disorder [43]. Intriguingly, Wu et al. also found that some CpG sites of epigenetic clocks are differentially methylated in SCZ patients [33]. Among these differentially methylated CpG sites (DMPs), 70–80% were located within the promoter regions. Furthermore, genes regulated by these DMPs displayed differential expression in SCZ patients and involved in the SCZ-related biological processes, such as immune dysregulation and neurological dysplasia [33]. These findings suggest that epigenetic clocks might be mediated by the dysregulation of pathophysiological processes in SCZ [33], which may be a potential cause of the epigenetic age deceleration underlying the development of SCZ. However, some previous studies have found that patients with SCZ had an accelerated epigenetic age or no difference in epigenetic age compared with healthy controls [29–32, 44]. Multiple clinical variables, such as trauma history, sex, and antipsychotic treatment have been associated with epigenetic aging processes [18, 35, 44], which may account for these inconsistent results and therefore should be taken into account in the future studies.

The current study demonstrated a significant effect of antipsychotic treatment on the Hannum and Levine clock, but not on the Horvath clock. Since each epigenetic clock is developed with different algorithms and captures distinct features of biological aging [15], these inconclusive findings among three epigenetic clocks are understandable. Horvath clock was developed with DNAm datasets from multiple tissues and development stages, measuring cellular aging independent of cell-type compositions [36]. Hannum clock was trained on blood samples, capturing more cell-extrinsic aging with moderate correlation with cell compositions [37]. Whereas the Levine clock was trained on the older adult population incorporating biological measures and capturing phenotypic age [38]. As Horvath clock is independent of cell-type compositions and cell-extrinsic biological measures [36], we supposed that the antipsychotic effect on accelerated epigenetic age in SCZ patients may be caused by the alterations of blood cell composition and other aging-related blood biomarkers. In line with our supposition, Talarico et al. recently found an accelerated epigenetic age in FSCZ patients after 10 weeks of treatment with risperidone. However, this result was not observed after adjusting for blood cell composition [42]. Furthermore, amounting evidence also has shown that antipsychotic treatment may influence the blood cell type compositions or other blood-based biomarkers [45, 46], which further support that the effects of antipsychotic treatment on epigenetic age may be biased by the blood cell composition. However, we cannot rule out the possibility that risperidone may accelerate epigenetic age through other biological processes. For example, risperidone treatment may change the methylation level of some CpG sites among epigenetic clocks, but this effect might be insufficient to be detected in the current study because of the short-term treatment.

Although SCZ-related genes were found to be regulated by the epigenetic clock and displayed abnormal expression in SCZ patients [33], the mechanism of epigenetic aging process implicated in the SCZ pathogenesis remains unclear. A recent study demonstrated a significant cross-sectional association between epigenetic age acceleration and psychosis severity that was measured by the Symptom Checklist 90 (SCL-90) psychotic domain [47]. Unlike this association finding, our analyses showed that epigenetic aging acceleration from all three epigenetic clocks was neither correlated with psychotic symptom severity (PANSS scores) nor symptom improvement (the percentage change on PANSS scores). In addition to the difference in symptom measures, the medicinal uses and substantially clinical heterogeneity in SCZ patients may partly account for this discrepancy.

We found no significant associations between epigenetic age acceleration and cognitive function in all participants at baseline. Our longitudinal analyses showed that baseline epigenetic age acceleration has an inverse association with the improvement of cognitive functions in some measures. However, this significant finding is not retained after multiple test corrections. This is not surprising given that the statistical power of our study is limited by the small sample size and a short-term follow-up. A recent large cross-sectional study investigated the association between two measures of epigenetic age acceleration (Horvath and Hannum) and three different neuropsychological tests in 4827 middle-aged participants from two independent cohorts, and only found a significant inverse association between Hannum AgeAccel and Word Fluency Test scores [48]. Another twin cohort study examined both cross-sectional and longitudinal associations (11.5-year) between four epigenetic clocks using Horvath, Hannum, Levine, and Grim algorithms and cognitive function using Trail Making Test (TMT) [20]. This twin study found that Horvath AgeAccel were correlated with cognitive decline longitudinally, but no association between epigenetic age acceleration and cognitive function at baseline [20]. Differences in the measures of epigenetic clocks and cognitive function in previous studies may account for the inconsistent results.

Only a few studies have investigated the relationship between epigenetic age acceleration and subcortical volume. A previous longitudinal study of 46 adolescent girls found Horvath AgeAccel related to reduced left hippocampal volume (4 years follow up) [23]. Similarly, two recent cross-sectional studies found a significant cross-sectional association between Hannum AgeAccel and reduced hippocampal volume, but this finding was not observed in Horvath’s clock [22, 25]. In our study, we failed to find any significant relationships between epigenetic age acceleration and subcortical volumes. This may also be related to different measures of epigenetic clocks and our small sample size as the cause of the discrepancy in cognitive function. Large replicate data with well-developed epigenetic clocks will be needed to draw a conclusion.

This study has several strengths. First, we investigated the epigenetic age acceleration in drug-naive FSCZ patients with a similar illness duration to exclude the potential confounding effects of medication and the pathophysiological process of SCZ on our results. Second, we analyzed the effect of medication on the epigenetic aging process in FSCZ through a longitudinal design of the cohort. Third, we implemented multiple epigenetic clocks that are designed with different algorithms and capture distinct epigenetic aging features, enabling a deeper understanding of various aging processes in SCZ. However, a major limitation of the present study should be noted. Our study has a relatively small sample size, which may limit the detective power of our study, accounting for the inconsistent findings from distinct epigenetic clocks analyses.

## Conclusions

This study provided evidence for the epigenetic age deceleration of Horvath clock in drug-naive FSCZ patients. Importantly, this longitudinal study demonstrated a potential effect of antipsychotic treatment on the epigenetic aging process in SCZ patients. Nevertheless, the inconclusive findings from three epigenetic clocks indicated that distinct epigenetic clocks are sensitive to different aspects of aging process. Large scale studies with long-term longitudinal designs and comprehensive epigenetic clock analyses are needed to confirm our findings and further advance our understanding of epigenetic aging progression in SCZ patients.

## Supplementary Information

**Additional file 1: Supplemental Table 1.** Demographics for Schizophrenia Patients and Healthy Controls.

## Data Availability

The datasets used and analysed during the current study available from the corresponding author on reasonable request.

## Abbreviations

SCZ: Schizophrenia
FSCZ: First-episode schizophrenia
DNAm: DNA methylation
PANSS: Positive and Negative Symptom Scale
DST: Digit Span Tests
VFT: Verbal Fluency Test
Stroop-W: the Stroop Words Tests
Stroop-C: the Stroop Colours Tests
Stroop-I: the Stroop Interference Tests
TMT: Trail Making Test
WCST-C: the Wisconsin Card Sorting Test-categories
WCST-P: the Wisconsin Card Sorting Test-perseverative errors
AgeAccel: Age Acceleration Residual
