# Supplementary material for: Epigenetic clock analysis of blood samples in drug-naive first-episode schizophrenia patients
Source: BMC Psychiatry. 2023 Jan 17;23:45. doi: 10.1186/s12888-023-04533-1 (PMC9843886; doi:10.1186/s12888-023-04533-1)
Supplement: Supplementary file 1 — Additional file 1: Supplemental Table 1. Demographics for Schizophrenia Patients and Healthy Controls. [file 12888_2023_4533_MOESM1_ESM.docx]

**Supplemental Table1 Demographics for Schizophrenia Patients and Healthy Controls**

| **Variables** | **SCZ** | | **Controls** | | **Analyses** | |
| --- | --- | --- | --- | --- | --- | --- |
|  | Mean | SD | Mean | SD | t-statistics | P value |
| Ages (years) | 25.00 | 4.95 | 24.76 | 4.56 | 0.22 | 0.83 |
|  | Male | Female | Male | Female | Chi-square | P value |
| Sex | 25 | 13 | 25 | 13 | 0.00 | 1 |
|  | Yes | No | Yes | No |  |  |
| Tobacco use (y/n) | 8 | 30 | 8 | 30 | 0.00 | 1 |
| Illness duration (Month) | 8.37 | 2.72 |  |  |  |  |
| Total PANSS Score | 93.21 | 11.46 |  |  |  |  |
| Positive PANSS Score | 25.82 | 3.59 |  |  |  |  |
| Negative PANSS Score | 18.63 | 5.64 |  |  |  |  |
| General PANSS Score | 93.21 | 11.46 |  |  |  |  |
